# Comparison of Intermolecular Halogen...Halogen Distances in Organic and Organometallic Crystals

**DOI:** 10.3390/ijms241511911

**Published:** 2023-07-25

**Authors:** Olga V. Grineva

**Affiliations:** Chemistry Department, Moscow M. V. Lomonosov State University, 119991 Moscow, Russia; ovg@phys.chem.msu.ru

**Keywords:** carbon–halogen group, metal–halogen group, halogen aggregation, van der Waals radii, Gaussian function

## Abstract

Statistical analysis of halogen...halogen intermolecular distances was performed for three sets of homomolecular crystals under normal conditions: C–Hal1...Hal2–C distances in crystals consisting of: (i) organic compounds (set Org); (ii) organometallic compounds (set Orgmet); and (iii) distances M1–Hal1...Hal2–M2 (set MHal) (in all cases Hal1 = Hal2, and in MHal M1 = M2, M is any metal). When analyzing C–Hal...Hal–C distances, a new method for estimating the values of van der Waals radii is proposed, based on the use of two subsets of distances: (i) the shortest distances from each substance less than a threshold; and (ii) all C–Hal...Hal–C distances less than the same threshold. As initial approximations for these thresholds for different Hal, the *R*_agg_ values previously introduced in investigations with the participation of the author were used (*R*_agg_ values make it possible to perform a statistical assessment of the presence of halogen aggregates in crystals). The following values are recommended in this work to be used as universal values for crystals of organic and organometallic compounds: *R*_F_ = 1.57, *R*_Cl_ = 1.90, *R*_Br_ = 1.99, and *R*_I_ = 2.15 Å. They are in excellent agreement with the results of some other works but significantly (by 0.10–0.17 Å) greater than the commonly used values. For the Orgmet set, slightly lower values for *R*_I_ (2.11–2.09 Å) were obtained, but number of the C–I...I–C distances available for analysis was significantly smaller than in the other subgroups, which may be the reason for the discrepancy with value for the Org set (2.15 Å). Statistical analysis of the M–Hal...Hal–M distances was performed for the first time. A Hal-aggregation coefficient for M–Hal bonds is proposed, which allows one to estimate the propensity of M–Hal groups with certain M and Hal to participate in Hal-aggregates formed by M–Hal...Hal–M contacts. In particular, it was found that, for the Hg–Hal groups (Hal = Cl, Br, I), there is a high probability that the crystals have Hg–Hal...Hal–Hg distances with length ≤ *R*_agg_.

## 1. Introduction

The role played by intermolecular contacts between halogen atoms (halogen...halogen contacts or Hal...Hal for short) in the formation of crystal structures of both organic [1,2] and organometallic [3,4] compounds is well known. This role is often reduced to considering only the shortest (shorter than the sum of van der Waals radii, i.e., 2*R*_Hal_ for identical Hal) contacts that can be described as a halogen bond [5]. Meanwhile, it was noted for the first time in [6] that the grouping of several halogen atoms can play an important role in the formation of crystal structures, even if not all Hal...Hal distances between neighboring atoms are shortened compared with 2*R*_Hal_. This phenomenon, for which we later proposed the name “aggregation of halogen atoms” or “Hal-aggregation”, was considered in a series of our works [7,8,9,10,11,12] with a number of examples.

Halogen atoms are not among the most common in protein or nucleic acid molecules; nevertheless, even almost 20 years ago in the PDB [13] (July 2004 release), 964 C–Hal bonds were found [14] in proteins and 321 in nucleic acids (for both sets Hal = Cl, Br, I). At the same time, halogenation of drugs often increases membrane binding and permeation [15], leading to the desired steric and conformational effects when interacting with the target site [16], so it is not surprising that the proportion of halogenated compounds among drugs is quite large (about 25% in DrugBank [17] according to [18]). In addition, intermolecular contacts involving halogen atoms contribute to the stabilization of protein-ligand complexes; therefore, plenty of works have been devoted to their study (e.g., [19,20,21,22]).

However, regardless of whether researchers consider only short Hal...Hal contacts or Hal-aggregates or halogen bonds in different type of substances, the starting point in the analysis is the values of the van der Waals radii of halogen atoms (*R*_Hal_). There have been a large number of works devoted to both the actual determination of *R*_Hal_ [23,24,25,26,27,28,29,30] and the discussion of values obtained by various methods [31]. In 2020, to determine the van der Waals radii of atoms in crystals, the authors [30] created arrays of distances, the longest of which obviously greatly exceeded the sum of van der Waals radii, and then they described them using Gaussian functions. An approach based on the use of several Gaussian functions to describe distance distributions in crystals was proposed by me a little later [32], independently of the authors [30].

In [30], the authors considered distances in crystals with molecules containing only H(D), B, C, N, O, F, Si, P, S, Cl, As, Se, Br, and I atoms. If the values of the van der Waals radii are constant in any environment, then the indicated restriction should not have a significant effect on the result. At the same time, without additional research, it cannot be ruled out that some differences in the structure of crystals consisting of molecules of only organic compounds or of molecules of organometallic compounds [33] may also affect the average values of certain intermolecular contacts.

Therefore, one of the purposes of this work was to compare the distributions of intermolecular distances Hal...Hal in two sets of crystals: those consisting of molecules of organic compounds (Org) and those of molecules of organometallic compounds (Orgmet). In addition, the statistical analysis of intermolecular metal–halogen...halogen–metal distances was of significant interest because of very scarce information about these distances in the literature.

## 2. Results

The procedure for the formation of different Hal...Hal distance sets that were used to perform statistical analysis is described in detail in Section 4. Table 1 provides information about the number of substances, the molecules of which have C–Hal bonds, for the sets Org and Orgmet. The proportions of these substances making up of the total number of crystals in the Org (138,916) and Orgmet (76,439) sets that satisfy all search conditions were also determined, except for the presence of a certain C–Hal bond, i.e., how common the substances with such bonds are. It can be seen that this fraction is the maximum for crystals with a C–Cl bond in the Org set (10.6%) and the minimum for crystals with a C–I bond in the Orgmet set (0.3%).

Next, in the formed sets, the C–Hal...Hal–C intermolecular distances were calculated for the same Hal atoms (*d*). In previous works [9], it was shown that the length of the Hal...Hal contacts involved in halogen aggregation usually does not exceed *R*_agg_ = 2*R*_Hal_ + 0.5 Å; i.e., in statistical studies (without a detailed analysis of aggregates in each substance), this quantity may be used to estimate the number of structures containing Hal-aggregates. In [9], *R*_F_ = 1.40 [27], *R*_Cl_ = 1.90 [26], *R*_Br_ = 1.97 [28], and *R*_I_ = 2.14 Å [28] were used; therefore, the upper limit *d* (*d*_max_) was initially determined in this work as 3.300, 4.300, 4.440, and 4.780 Å for the F...F, Cl...Cl, Br...Br, and I...I distances, respectively. The number of substances containing such distances is also indicated in Table 1.

It turned out that, according to this criterion, the fraction of substances containing Hal-aggregates is systematically higher in the Orgmet set than in Org, with the greatest difference (by 22.6%) being observed for F-aggregates and the smallest difference for I-aggregates (3.8%).

## 3. Discussion

### 3.1. Analysis of the Shortest C–Hal...Hal–C Distances

The shortest C–Hal...Hal–C distances of various types for crystals of organic compounds are reported in Table 2. The analysis of the adequacy of such distances is usually not carried out, although the almost twofold reduction in distances compared to 2*R*_Hal_ apparently indicates errors in the determination of structures. In this work, the following approach was used to assess the adequacy of the shortest distances. All distances of each type were sorted by increase, and if the difference between the previous and next contact was more than 0.1 Å, then the previous contact was classified as unrealistically short. The presence of too short Hal...Hal distances may be associated with general errors in the determination of the structure; therefore, not only these short distances but also all distances in the corresponding substances (records) were excluded from further analysis. It turned out that there was no need to remove substances only from subsets of contacts I...I. In the remaining subsets, the entries PUCREL, FFMXZP, JODVAZ, VAWNUE, MIWHOP01, and XACXEE in the Org set and TUKNOC, KUSMOD, and CUCNUM in the Orgmet set were deleted. The number and range of distances used in further analysis are shown in Table 3. The results obtained below indicate that the previously used value *R*_F_ = 1.40 Å is underestimated; therefore, the sample for C–F...F–C distances was also extended for this type of distance in Table 3 and has two rows.

It can be seen that, in the corrected arrays, the reduction in the shortest distances compared to 2*R*_Hal_ (proposed in [26,27,28]) does not exceed ~20%, which seems reasonable.

### 3.2. Analysis of Distance Distributions

**Figure 1:**
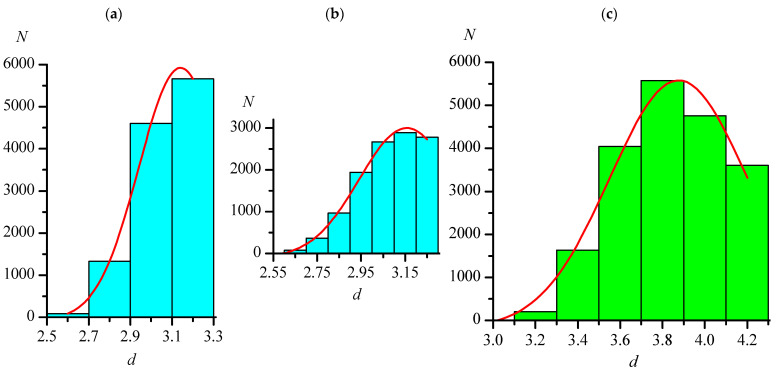

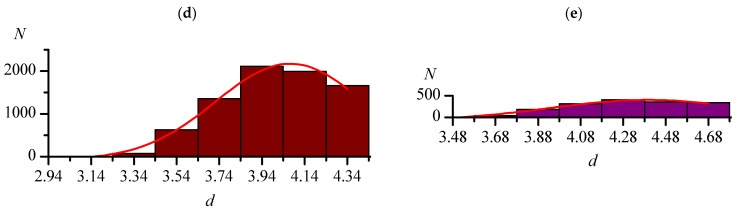
Distributions of the lengths (*d*, Å) of intermolecular distances C–Hal...Hal–C in crystals of the Org set, not exceeding 3.300, 4.300, 4.440, and 4.780 Å for F...F ((**a**) has a step of 0.2 Å, (**b**) has a step 0.1 Å)), Cl...Cl (**c**), Br...Br (**d**), and I...I (**e**), respectively. The red lines are the approximations by the Gaussian function.

**Figure 2:**
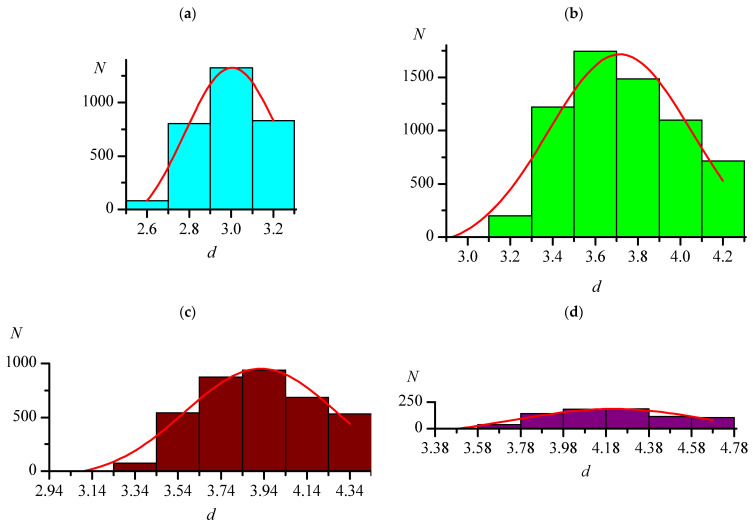
Distributions of the lengths (*d*, Å) of the shortest intermolecular distances C–Hal...Hal–C (one of each substance) in crystals of the Org set: F...F (**a**), Cl...Cl (**b**), Br...Br (**c**), I...I (**d**). The red lines are the approximations by the Gaussian function.

Often, when determining van der Waals radii, only the shortest distances are considered. However, van der Waals radii are most valuable when they allow for estimation of not the shortest but the most probable non-valence distances. In this paper, for each type of distances C–Hal...Hal–C in each of the two main sets Org and Orgmet, two variants of distance arrays were analyzed. The first one included all distances of the corresponding type within *d*_max_ in the selected substances, while the second included the one shortest distance of the corresponding type from each selected substance; thus, the longest distances in the second array also did not exceed *d*_max_. For these arrays of distances, histograms were constructed, examples of which for the Org set are shown in Figure 1 and Figure 2.

The maxima on all histograms were described by Gaussian function (red lines in Figure 1 and Figure 2). It should be noted that, when the histogram step changes, the view of histogram changes to some extent (for example, Figure 1a,b shows the distributions of the F...F distances with steps of 0.2 and 0.1 Å), but the position of the maximum when described by the Gaussian function remains almost constant (see Table 4 and Table 5). In Figure 1a, the position and the very presence of the maximum do not seem obvious, which, as noted earlier, is most likely a consequence of the initially chosen *R*_agg_ value for the F...F distances being underestimated. Therefore, additional arrays with *d*_max_ = 3.50 Å were analyzed for this type of distance. Table 4 shows that, in this case, as for other types of distances, the distribution parameters depend very little on the histogram step.

Table 6 shows the differences between the positions of the maxima of the Gaussian functions for different options used for histograms.

The results for I…I contacts in the Orgmet set do not quite match the trends for other types of contacts in several cases. Perhaps this outcome is due to the significantly smaller number of I…I contacts in this set, especially for the sample of the shortest contacts.

In the Org set, the differences in the positions of the maxima for histograms with steps of 0.2 and 0.1 Å do not exceed 0.011 Å. The same maximum difference appears in the Orgmet set if the results for contacts I…I are not considered. Thus, the differences in the positions of the maxima between Org and Orgmet can be considered significant if they exceed 0.01 Å.

It turns out that the positions of the maxima for the shortest contacts of all types in Orgmet correspond to significantly shorter distances than in Org: the contraction is in the range of 0.023–0.055 Å for contacts F…F, Cl…Cl, and Br…Br. For the same contacts from the All arrays, a different pattern is observed: for F…F, on average, shorter (by 0.050–0.058 Å) contacts exist in the Org set, Cl…Cl contacts are also shorter in Org but only by 0.013–0.014 Å, and the Br…Br contacts are on average longer (by 0.017–0.022 Å) in Org than in Orgmet.

As expected, the maxima for First contacts correspond to shorter distances than the maxima for All for all types of distances in both sets (Org and Orgmet), while in its meaning, the sum of van der Waals radii should be greater than the maximum values for First and less than the maximum values for All.

The expression (*x*_all_ + *x*_first_)/4 was used to estimate the value of the van der Waals radii. It turns out (Table 7) that the values obtained in this way are in excellent (within 0.01 Å) agreement with each other, both for different histogram spacing and for the Org and Orgmet sets. An exception is the discrepancy in the *R*_I_ estimates for Org and Orgmet, which, as noted above, may be due to an insufficient number of contacts in the C–I… I–C sample for Orgmet.

Table 8 compares the results of determining the van der Waals radii in this work with some data from the literature.

The values obtained in this work are in good agreement with the data [30] obtained using a similar technique. At the same time, it is important to note the good agreement between the values for Cl, Br, and I with the results [26,27,28], which were obtained by another method and which were previously used to estimate statistically the values of *R*_agg_. Thus, the previously obtained data on halogen aggregation involving these atoms remain relevant, while the data on F-aggregation can be revised considering the new value of *R*_F_.

Thus, the obtained results indicate that the following van der Waals radii for halogen atoms bonded to a carbon atom can be recommended as unified values for crystals of organic and organometallic compounds under normal conditions: *R*_F_ = 1.57, *R*_Cl_ = 1.90, *R*_Br_ = 1.99, and *R*_I_ = 2.15 Å. It makes sense to clarify the value of *R*_I_ in crystals of organometallic compounds when data for a larger number of structures become available.

### 3.3. The M–Hal…Hal–M Distances

Halogen bonds involving M–Hal groups have been the subject of many studies [3,4,34,35,36]. However, as a rule, contacts of halogen atoms from such groups have been considered either with other elements or with halogen atoms that do not form M–Hal bonds. Contacts M–Hal…Hal–M have rarely been noted by researchers [37]. Therefore, one of the goals of this work was to statistically analyze the M1–Hal1…Hal2–M2 distances. In this case, as in the analysis of the C–Hal1…Hal2–C distances, only the distances between the same atoms (Hal1 = Hal2, M1 = M2) under normal conditions were considered. The rules for selecting substances for the MHal set are described in more detail in Section 4.

Data on the number of different metal elements (M) with M–Hal bonds, according to the CSD [38] search, are provided in Table 9. Detailed information about the number of symmetrically independent bonds of each M–Hal type is given in Table 10. It can be seen that, among the studied substances, some types of M–Hal bonds are very rare. Therefore, in addition to the number of metal elements that have at least one particular M–Hal bond in all crystals, the numbers of M with more than 20 and more than 40 symmetrically independent M–Hal bonds in total are presented in Table 9.

Table 10 shows the statistical data characterizing all the M–Hal groups found in CSD (under normal conditions of crystal study): *N*_M–Hal_ is the number of symmetrically independent bonds; *N*_Hal…Hal_ is the number of distances M–Hal…Hal–M ≤ *R*_agg_, as well as the Hal-aggregation coefficient for M–Hal bonds, which is proposed in this work to estimate the propensity of M–Hal groups of a certain type to participate in aggregates formed by M–Hal…Hal–M contacts, *k*_MHal-agg_ = *N*_Hal…Hal_/*N*_M–Hal_. The higher the *k*_MHal-agg_ value is, the more likely it is that the M–Hal group with specific M and Hal forms at least one M–Hal…Hal–M contact with length ≤ *R*_agg_. In this part of the work, when calculating *R*_agg_ = 2*R*_Hal_ + 0.5 Å, the *R*_Hal_ values obtained in the previous stage as a result of the analysis of the C–Hal…Hal–C distances (Section 3.2) were used, namely: *R*_F_ = 1.57, *R*_Cl_ = 1.90, *R*_Br_ = 1.99, and *R*_I_ = 2.15 Å.

**Figure 3:**
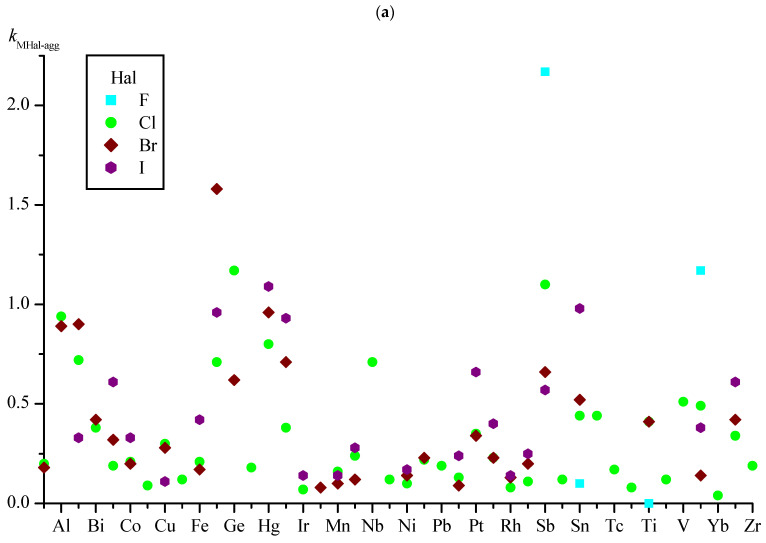

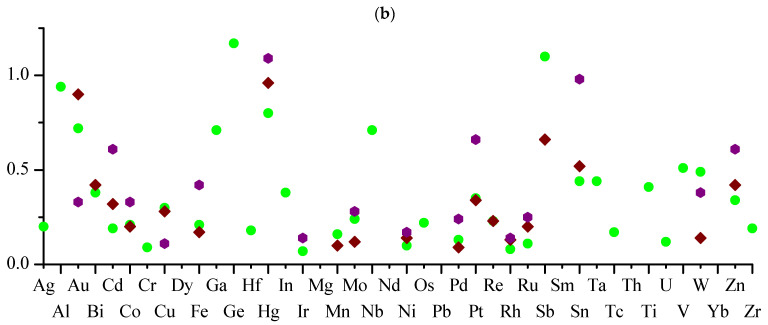
Values of Hal-aggregation coefficients for M–Hal bonds (*k*_MHal-agg_) for metals with more than 20 M–Hal bonds in the set (**a**) and more than 40 bonds (**b**).

Obviously, the *k*_MHal-agg_ coefficient has a predictive value if the number of symmetrically independent M–Hal bonds in the training set is sufficiently large. The smallest numbers of such bonds were found for the M–F groups; therefore, the *N*_M–Hal_ boundaries in the analysis of *k*_MHal-agg_ were chosen, considering the available *N*_M–F_. The graphs illustrating the changes in *k*_MHal-agg_ depending on M and Hal show values for metals having more than 20 symmetrically independent M–Hal bonds in the set (Figure 3a) and more than 40 bonds (Figure 3b) (for convenience, the abscissa scales on Figure 3a,b are made identical; elements M are listed in alphabetical order).

In the set under consideration, when the condition *N*_M–Hal_ > 20 is fulfilled (Figure 3a), the maximum and minimum values of *k*_MHal-agg_ correspond to bonds involving fluorine. The largest value of *k*_MHal-agg_ (2.17) is for the Sb–F group and the smallest (0) for the Ti–F group; i.e., the groups of Sb–F participate on average in more than two symmetrically independent distances of Sb–F...F–Sb, while the Ti–F groups are not at all inclined to form Ti–F...F–Ti contacts. It should be noted that the number of symmetrically independent groups of M–F in both cases is not too large (35 each), and as their number increases, the values of *k*_MHal-agg_ can notably change.

In the subset with *N*_M–Hal_ > 40 (Figure 3b), the largest value of *k*_MHal-agg_ (1.17) is for the Ge–Cl group, while the value of Hg–I is close to it (1.09). In general, for the Hg–Hal groups (Hal = Cl, Br, I), the *k*_MHal-agg_ values are high (0.80–1.09), while the numbers of such groups in the set is 190 or more (Table 10); i.e., with high probability, one can expect the presence of substances of distances of Hg–Hal...Hal–Hg with length ≤ *R*_agg_ for these Hal.

To estimate the parameters of the distributions of the M–Hal...Hal–M distances, the same approach was used as for the C–Hal...Hal–C distances. It should be noted that the smallest number of C–Hal...Hal–C distances is in the First subset of the Orgmet set for C–I...I–C (140). In the MHal set, there is about the same number of values (124) in the All subset for M–F...F–M distances, and in the First subset for distances of the same type, it is several times less (33). Accordingly, the error in parameters describing these distances can be high, especially in the First subset, as evidenced in particular by the low correlation coefficient for it (*r*^2^ = 0.702). The distances of M–Hal1...Hal2–M (Hal1 = Hal2 = Cl, Br, I) were described using Gaussian functions with two step sizes (0.1 and 0.2 Å). In this case, as in the analysis of the C–Hal...Hal–C distances, the position of the maxima of the functions changed very little; therefore, Table 11 lists only the parameters of the distributions obtained with a step of 0.2 Å.

Considering that the Hal atoms in the M–Hal groups can coexist with large ligands around M, which hinder the approach of the same groups from neighboring molecules, it is not surprising that the positions of the maximum exceed 2*R*_Hal_, even in the First subsets. Somewhat surprisingly, for M–Cl...Cl–M, the *x*_All_ value is almost insignificant (by 0.026 Å) but is less than *x*_First_. Formally, for the distances of M–F...F–M, the same effect is observed and is much larger in magnitude; however, it could be due to the small number of these distances in the set, and, accordingly, the inaccuracy of the obtained parameters. For the M–Br...Br–M and M–I...I–M distances, the values of *x*_m_ in the All subset are expectedly greater than in the First subset, but the difference of *x*_all_–*x*_first_ for M–Br...Br–M is notably smaller (0.053 Å) on average than for the C–Hal...Hal–C distances (Table 6).

## 4. Materials and Methods

### 4.1. General Procedure for the Formation of Hal...Hal Distance Sets

For the selection of crystalline substances, the Cambridge Structural Database (CSD) [38], version 5.43 (November 2021), +3 updates was used. The search was carried out with the ConQuest program [39], using combinations of several conditions.

Small halogen-containing molecules (CCl_4_, CHCl_3_, etc.) often occur in crystals as solvate molecules, and their position and/or orientation is often disordered, which is not always accurately described when determining the structure. Therefore, to increase the accuracy of the results, not only all records with disorder noted in CSD were excluded from consideration but also the structures of only homomolecular crystals (crystals consisting of identical molecules; the search condition in ConQuest NRes = 1) were considered.

Since the goal of this work was to analyze distances involving only terminal halogen atoms, when drawing the C–Hal or M–Hal fragments, the presence of only one bond was specified for Hal.

The specificity of the formation of crystals of polymeric compounds can lead to a difference in the distributions of the lengths of interatomic distances in them compared to crystals of nonpolymeric compounds; therefore, the corresponding entries were also excluded from consideration.

Temperature and pressure can affect the parameters of intermolecular contacts, so room temperature and normal pressure were other search conditions (records with the “pressure” field were excluded).

The Best room temperature list [40] was used to determine the best studies within the same family (records with the same letter part of the reference code).

The conditions listed above, supplemented by the presence of 3D coordinates (which was necessary for further distance calculations) and the absence of CSD error warnings, were used for searches.

### 4.2. Additional Terms for Particular Sets

To form a set of distances of C–Hal...Hal–C existing in crystals of organic compounds (Org), a search was carried out among records classified in CSD as Organics. Accordingly, to form a set of distances observed in crystals of organometallic compounds (Orgmet), the search was performed among records classified in the CSD as Organometallic. To form a set of distances of M–Hal...Hal–M, when drawing, M–Hal M was taken as ‘any metal’, and the search was performed on all records (i.e., without choosing Organics or Organometallic).

## 5. Conclusions

The values of the van der Waals radii of halogen atoms obtained in this work are in excellent agreement with the results of Chernyshov et al. [30] but significantly (by 0.10–0.17 Å) greater than the commonly used values of Bondi [25].

The positions of the maxima of the Gaussian functions, describing the shortest distances of C–Hal...Hal–C in each substance, are shifted to shorter distances in the set of homomolecular crystals of organometallic compounds compared to crystals of organic compounds. At the same time, the values of the van der Waals radii proposed considering the characteristics of the two distributions of contacts C–Hal... Hal–C (the shortest in each substance and all less than the threshold while somewhat greater than *R*_Hal_) are almost the same in these two sets of substances, with the exception of radius I. The difference in values of radius I may be attributed to a significantly smaller number of C–I...I–C contacts in the set of crystals of organometallic compounds.

An extensive statistical analysis of the M–Hal...Hal–M distances has been performed for the first time. The Hal-aggregation coefficient for M–Hal bonds was proposed and calculated for all M–Hal groups presented in the MHal set, making it possible to estimate the propensity of M–Hal groups with certain M and Hal to participate in Hal-aggregates formed by M–Hal...Hal–M contacts.

## Figures and Tables

**Table 1:** Results of search in the CSD for substances with C–Hal bonds and C–Hal...Hal–C distances satisfying the Hal-aggregation statistical criterion proposed in [9] (*N* is the number of substances with C–Hal bonds, *N*_tot_ is the total number of substances in the sets Org and Orgmet, and *N*_agg_ is the number of substances with distances satisfying the Hal-aggregation criterion with *R*_Hal_ from [26,27,28]).

C–Hal Bonds					C–Hal...Hal–C Distances				
Type	Org		Orgmet		Type	Org		Orgmet	
	*N*	*N*/*N*_tot,_ %	*N*	*N*/*N*_tot,_ %		*N*_agg_	*N*_agg_/*N*_,_ %	*N*_agg_	*N*_agg_/*N*_,_ %
C–F	9682	7.0	3841	5.0	C–F...F–C	4069	42.0	2481	64.6
C–Cl	14,703	10.6	2685	3.5	C–Cl...Cl–C	7729	52.6	1592	59.3
C–Br	9960	7.2	1359	1.8	C–Br...Br–C	4544	45.6	804	59.2
C–I	1792	1.3	226	0.3	C–I...I–C	1042	58.1	140	61.9

**Table 2:** The CSD refcodes for the five shortest C–Hal...Hal–C distances in homomolecular crystals of organic compounds at normal pressure and room temperature (*d* is the contact length in Å and in % with respect to 2.80, 3.80, 3.94, and 4.28 Å (2*R*_Hal_ [26,27,28]) for F...F, Cl...Cl, Br...Br, and I...I, respectively).

F...F	*d*		Cl...Cl	*d*		Br...Br	*d*		I...I	*d*	
	Å	%		Å	%		Å	%		Å	%
PUCREL	1.498	54	MIWHOP01	2.835	75	XACXEE	2.992	76	WEWGAJ	3.567	83
PUCREL	1.556	56	PORWIC	3.050	80	DBRDOX	3.148	80	QASXOA	3.602	84
FFMXZP	1.774	63	ZIVKIY	3.064	81	KOVTEX	3.244	82	NUFCIC	3.616	84
JODVAZ	2.181	78	CLMPMO	3.079	81	HEGCII	3.256	83	DEQKUI	3.618	84
VAWNUE	2.187	78	TIJVEN	3.094	81	ADOPOB	3.267	83	VALRAD	3.644	85

**Table 3:** Characteristics of the C–Hal...Hal–C intermolecular distances in initial arrays (Initial) and used in statistical analysis (In analysis).

	*R*_agg_, Å	Org					Orgmet				
		Initial		In Analysis			Initial		In Analysis		
		Number	Shortest, Å	Number	Shortest, Å	/2*R*_Hal_, %	Number	Shortest, Å	Number	Shortest, Å	/2*R*_Hal_, %
C–F...F–C	3.300	14,533	1.498	14,514	2.503	89.4	10,797	1.960	10,793	2.353	84.0
	3.500	21,013		20,985			16,456		16,451		
C–Cl... Cl–C	4.300	22,361	2.835	22,359	3.050	80.3	4839	2.096	4828	3.028	79.7
C–Br... Br–C	4.440	9501	2.992	9472	3.148	79.9	1833	1.954	1821	3.174	80.6
C–I... I–C	4.780	2177	3.567	2177	3.567	83.3	396	3.648	396	3.648	85.2

**Table 4:** The main parameters for describing the distribution of the lengths of all C–Hal...Hal–C intermolecular distances less than the *d*_max_ values in crystals of the Org and Orgmet sets using the Gaussian function with different histogram steps (*x*_m_ is the position of the maximum, *r* is the correlation coefficient).

Distances	F...F				Cl...Cl ≤4.300 Å		Br…Br ≤ 4.440 Å		I…I ≤ 4.780 Å	
	≤3.300 Å		≤3.500 Å							
Step, Å	0.1	0.2	0.1	0.2	0.1	0.2	0.1	0.2	0.1	0.2
Org										
Total	14,514		20,985		22,359		9472		2177	
*x*_m_	3.156	3.141	3.242	3.239	3.878	3.879	4.067	4.066	4.384	4.388
*r* ^2^	0.997	1	0.979	0.987	0.980	0.987	0.980	0.990	0.967	0.983
Orgmet										
Total	10,793		16,451		4828		1821		396	
*x*_m_	3.199	3.186	3.300	3.289	3.891	3.893	4.050	4.044	4.409	4.262
*r* ^2^	0.999	0.9999	0.989	0.996	0.978	0.993	0.977	0.997	0.907	0.980

**Table 5:** The main parameters for describing the distribution of the lengths of the shortest intermolecular distances C–Hal…Hal–C (one of each substance) less than the *d*_max_ values in crystals of the Org and Orgmet sets using the Gaussian function with different histogram steps (*x*_m_ is the position of the maximum, *r* is the coefficient correlations).

Distances	F…F ≤ 3.500 Å		Cl…Cl ≤ 4.300 Å		Br…Br ≤ 4.440 Å		I…I ≤ 4.780 Å	
Org								
Total	4744		7728		4543		1042	
Step, Å	0.1	0.2	0.1	0.2	0.1	0.2	0.1	0.2
*x*_m_	3.028	3.039	3.730	3.729	3.926	3.927	4.232	4.228
*r* ^2^	0.911	0.922	0.912	0.938	0.937	0.964	0.905	0.952
Orgmet								
Total	2751		1591		803		140	
Step, Å	0.1	0.2	0.1	0.2	0.1	0.2	0.1	0.2
*x*	2.977	2.984	3.702	3.706	3.893	3.891	4.049	4.102
*r* ^2^	0.949	0.966	0.951	0.978	0.926	0.950	0.373	0.765

**Table 6:** Differences between the positions of the maxima (*x*) of the Gaussian functions for different variants of histograms for the C–Hal… Hal–C distances in the Org and Orgmet sets (All—all contacts less or equal *d*_max_, (Å); First—one shortest contact from each substance, 0.1 is a histogram with a step of 0.1 Å, and 0.2 is a histogram with a step of 0.2 Å).

Type of Hal…Hal	*d*_max_	*x*_0.2_–*x*_0.1_				*x*_Org_–*x*_Orgmet_				*x*_all_–*x*_first_	
		Org		Orgmet		0.1		0.2		0.2	
		All	First	All	First	All	First	All	First	Org	Orgmet
F…F	3.50	−0.003	0.011	−0.011	0.007	−0.058	0.051	−0.050	0.055	0.200	0.305
Cl…Cl	4.30	0.001	−0.001	0.002	0.004	−0.013	0.028	−0.014	0.023	0.150	0.187
Br…Br	4.44	−0.001	0.001	−0.006	−0.002	0.017	0.033	0.022	0.036	0.139	0.153
I…I	4.78	0.004	−0.004	−0.147	0.053	−0.025	0.183	0.126	0.126	0.160	0.160

**Table 7:** Estimation of the van der Waals radii of halogen atoms (Å) by the formula (*x*_all_ + *x*_first_)/4, where x is a position of maximum of Gaussian function used for description of the subset of the C–Hal…Hal–C distances (All—all contacts with ≤*d*_max_; First—one shortest contact from each substance ≤ *d*_max_; Org—homomolecular crystals of compounds; Orgmet—homomolecular crystals of organometallic compounds).

Hal	Org		Orgmet	
Step, Å	0.1	0.2	0.1	0.2
F	1.57	1.57	1.57	1.57
Cl	1.90	1.90	1.90	1.90
Br	2.00	2.00	1.98	1.98
I	2.15	2.15	2.11	2.09

**Table 8:** Comparison of the van der Waals radii of halogen atoms (Å) obtained in this work with some literature data.

Hal	This Work	Previous Values				
		Pauling [23]	Kitaigorodskii [24]	Bondi [25]	Zefirov et al.	Chernyshov et al. [30]
F	1.57	1.35		1.47	1.40 [27]	1.55
Cl	1.90	1.80	1.78	1.75	1.90 [26]	1.91
Br	2.00 (Org), 1.98 (Orgmet)	1.95	1.95	1.85	1.97 [28]	2.00
I	2.15 (Org), 2.10 (Orgmet)	2.15	2.1	1.98	2.14_5_ [28]	2.17

**Table 9:** Number of different metal atoms (M) forming M–Hal bonds in homomolecular crystals, including those with >20 and >40 symmetrically independent bonds with a specific Hal.

Hal	F	Cl	Br	I
At least one M–Hal	29	64	53	57
>20 M–Hal	4	41	27	21
>40 M–Hal	0	35	19	16

**Table 10:** Statistical data for the M–Hal groups (*N*_M–Hal_ is the number of symmetrically independent bonds; *N*_Hal…Hal_ is the number of distances M1–Hal1…Hal2–M2 ≤ *R*_agg_; *k*_MHal-agg_ is the statistical Hal-aggregation coefficient for M–Hal bonds, M1 = M2, Hal1 = Hal2; *R*_Hal_ values determined in this work were used to calculate *R*_agg_, *k*_MHal-agg_ = *N*_Hal…Hal_/*N*_M–Hal_).

	M	F			Cl			Br			I		
		*N*_M–Hal_	*N*_Hal…Hal_	*k*_MHal-agg_	*N*_M–Hal_	*N*_Hal…Hal_	*k*_MHal-agg_	*N*_M–Hal_	*N*_Hal…Hal_	*k*_MHal-agg_	*N*_M–Hal_	*N*_Hal…Hal_	*k*_MHal-agg_
1	Ag	0	0	−	83	17	0.20	38	7	0.18	14	0	0
2	Al	3	1	0.33	244	229	0.94	38	34	0.89	18	27	1.50
3	Au	0	0	−	500	361	0.72	91	82	0.90	70	23	0.33
4	Ba	0	0	−	1	0	0	0	0	−	3	0	0
5	Be	0	0	−	8	3	0.38	2	0	0	3	2	0.67
6	Bi	3	0	0	96	36	0.38	47	20	0.42	20	14	0.70
7	Ca	0	0	−	5	1	0.20	4	0	0	5	0	0
8	Cd	0	0	−	263	50	0.19	78	25	0.32	195	119	0.61
9	Ce	0	0	−	5	0	0	0	0	−	0	0	−
10	Co	4	0	0	583	121	0.21	102	21	0.20	87	29	0.33
11	Cr	0	0	−	148	14	0.09	15	0	0	12	0	0
12	Cs	0	0	−	2	0	0	0	0	−	0	0	−
13	Cu	2	0	0	1465	437	0.30	460	129	0.28	229	26	0.11
14	Dy	0	0	−	40	5	0.12	6	0	0	0	0	−
15	Er	0	0	−	9	1	0.11	0	0	−	1	0	0
16	Eu	0	0	−	11	3	0.27	0	0	−	1	0	0
17	Fe	2	0	0	409	86	0.21	52	9	0.17	89	37	0.42
18	Ga	1	3	3.00	165	117	0.71	26	41	1.58	27	26	0.96
19	Gd	0	0	−	15	3	0.20	1	0	0	0	0	−
20	Ge	8	0	0	154	180	1.17	21	13	0.62	13	11	0.85
21	Hf	10	0	0	97	17	0.18	8	4	0.50	5	2	0.40
22	Hg	0	0	−	461	367	0.80	190	182	0.96	245	267	1.09
23	Ho	0	0	−	10	3	0.30	0	0	−	0	0	−
24	In	2	2	1.00	99	38	0.38	31	22	0.71	28	26	0.93
25	Ir	0	0	−	461	32	0.07	16	0	0	65	9	0.14
26	K	0	0	−	0	0	−	2	0	0	5	0	0
27	La	0	0	−	13	3	0.23	3	0	0	2	0	0
28	Li	0	0	−	7	0	0	7	0	0	5	0	0
29	Lu	0	0	−	8	0	0	1	0	0	2	0	0
30	Mg	0	0	−	12	3	0.25	25	2	0.08	5	0	0
31	Mn	1	1	1.00	242	39	0.16	99	10	0.10	22	3	0.14
32	Mo	6	2	0.33	310	75	0.24	101	12	0.12	97	27	0.28
33	Na	0	0	−	1	0	0	2	0	0	6	0	0
34	Nb	3	0	0	209	149	0.71	9	17	1.89	4	0	0
35	Nd	0	0	−	25	3	0.12	1	0	0	1	0	0
36	Ni	2	0	0	389	38	0.10	257	36	0.14	53	9	0.17
37	Np	0	0	−	3	0	0	0	0	−	0	0	−
38	Os	1	0	0	153	34	0.22	39	9	0.23	20	4	0.20
39	Pb	0	0	−	32	6	0.19	12	0	0	19	6	0.32
40	Pd	7	2	0.28	2133	282	0.13	281	25	0.09	227	55	0.24
41	Pr	0	0	−	16	3	0.19	0	0	−	0	0	−
42	Pt	4	2	0.50	1530	533	0.35	132	45	0.34	248	164	0.66
43	Pu	0	0	−	1	0	0	0	0	−	1	2	2.00
44	Rb	0	0	−	1	0	0	1	0	0	0	0	−
45	Re	2	0	0	670	153	0.23	271	63	0.23	40	16	0.40
46	Rh	3	0	0	619	51	0.08	45	6	0.13	79	11	0.14
47	Ru	2	0	0	1533	174	0.11	74	15	0.20	81	20	0.25
48	Sb	35	76	2.17	205	226	1.10	65	43	0.66	28	16	0.57
49	Sc	0	0	−	8	2	0.25	0	0	−	1	0	0
50	Sm	0	0	−	24	3	0.12	2	0	0	19	1	0.05
51	Sn	21	2	0.10	1080	473	0.44	172	90	0.52	90	88	0.98
52	Sr	0	0	−	0	0	−	2	2	1.00	4	0	0
53	Ta	3	1	0.33	208	91	0.44	10	4	0.40	3	0	0
54	Tb	0	0	−	6	1	0.17	0	0	−	0	0	−
55	Tc	0	0	−	69	12	0.17	8	1	0.12	1	0	0
56	Th	0	0	−	26	2	0.08	1	0	0	1	1	1.00
57	Ti	35	0	0	845	348	0.41	39	16	0.41	6	1	0.17
58	Tl	1	0	0	14	8	0.57	8	13	1.62	4	4	1.00
59	Tm	0	0	−	2	0	0	0	0	−	1	0	0
60	U	4	4	1.00	96	12	0.12	18	1	0.06	11	1	0.09
61	V	4	1	0.25	135	69	0.51	4	1	0.25	8	1	0.12
62	W	23	27	1.17	356	174	0.49	49	7	0.14	86	33	0.38
63	Y	0	0	−	20	0	0	0	0	−	2	1	0.50
64	Yb	0	0	−	26	1	0.04	4	0	0	10	0	0
65	Zn	2	0	0	953	322	0.34	262	109	0.42	188	115	0.61
66	Zr	5	0	0	579	108	0.19	13	8	0.62	14	5	0.36

**Table 11:** The main parameters for describing the length distribution of all intermolecular M–Hal...Hal–M distances (All) and the shortest ones in each substance (First) less than the *R*_agg_ values in crystals of the MHal set by the Gaussian function with a histogram step of 0.2 Å (*x*_m_ is the maximum position; *r* is the correlation coefficient; when calculating *R*_agg_, the *R*_Hal_ values determined in this work were used).

Distances	F...F ≤ 3.640 Å		Cl...Cl ≤ 4.300 Å		Br...Br ≤ 4.480 Å		I...I ≤ 4.800 Å	
Subset	All	First	All	First	All	First	All	First
Total	124	33	5554	3156	1127	653	1203	686
*x*_m_	3.300	3.500	4.083	4.109	4.218	4.165	4.593	4.449
*r* ^2^	0.998	0.702	0.996	0.995	0.9997	0.9997	0.998	0.994
